# The Role of the Cerebellum in Multiple Sclerosis-Related Fatigue and Disability

**DOI:** 10.3390/jcm14082840

**Published:** 2025-04-20

**Authors:** Nicola Manocchio, Ornella Argento, Michela Bossa, Barbara Spanò, Leonardo Pellicciari, Calogero Foti, Ugo Nocentini

**Affiliations:** 1 Physical and Rehabilitation Medicine, Department of Clinical Sciences and Translational Medicine, University of Rome Tor Vergata, 00133 Rome, Italy; bossa.michela@gmail.com (M.B.); foti@med.uniroma2.it (C.F.); u.nocentini@hsantalucia.it (U.N.); 2 Behavioural Neuropsychology, IRCCS “Santa Lucia” Foundation, 00179 Rome, Italy; argentornella15@gmail.com; 3 Neuroimaging Unit, IRCCS “Santa Lucia” Foundation, 00179 Rome, Italy; b.spano@hsantalucia.it; 4 IRCCS Istituto delle Scienze Neurologiche di Bologna, 40139 Bologna, Italy; leonardo.pellicciari@gmail.com

**Keywords:** multiple sclerosis, fatigue, modified fatigue impact scale, cerebellum, disability, magnetic resonance imaging, quality of life

## Abstract

**Background**: Fatigue is a prevalent and debilitating symptom in people with multiple sclerosis (pwMS), significantly impairing quality of life. While the cerebellum is traditionally associated with motor control, emerging evidence suggests its involvement in cognitive, emotional, and integrative functions. This study aimed to explore the relationship between fatigue components (physical, cognitive, and psychosocial), clinical disability, and cerebellar structural changes in pwMS acquired via magnetic resonance imaging (MRI). **Methods**: Participants of this cross-sectional study underwent clinical assessments for fatigue (Modified Fatigue Impact Scale) and disability (Expanded Disability Status Scale). Cerebellar volumes were measured using high-resolution MRI and voxel-based morphometry (VBM) to identify correlations between fatigue subdomains and specific cerebellar subregions. Statistical analyses included group comparisons and correlation tests. **Results**: Forty-four pwMS were included. Fatigued MS patients exhibited reduced sensorimotor cerebellar volumes compared to non-fatigued counterparts. Physical fatigue correlated negatively with sensorimotor cerebellum volume, while cognitive fatigue showed an inverse relationship with limbic cerebellum regions. Interestingly, psychosocial fatigue was positively associated with limbic cerebellum volume, contrary to initial hypotheses. Higher disability scores were linked to atrophy in cognitive and limbic cerebellar regions. **Conclusions**: The findings highlight the cerebellum’s multifaceted role in MS-related fatigue, with distinct subregions contributing to physical, cognitive, and psychosocial fatigue components. These results underscore the cerebellum’s critical function as a hub for motor, cognitive, and emotional integration. Future longitudinal studies incorporating objective measures and advanced imaging are essential to elucidate these relationships further and inform targeted therapeutic strategies for pwMS.

## 1. Introduction

Fatigue is defined as the inability to maintain force output over a period of time. Importantly, fatigue is distinct from weakness; it represents a specific symptom rather than a generalized reduction in strength [1]. Chaudhuri and Behan suggested the term “central fatigue” (CF) to highlight/describe the failure in performing/sustaining both mental (cognitive) and physical (motor) tasks requiring self-motivation [2]. Fatigue is one of the most frequent and debilitating symptoms reported by people with multiple sclerosis (pwMS), with a prevalence of around 80% [3]. This pervasive symptom significantly impairs activities of daily living (ADLs), including work productivity and overall quality of life (QoL) [4,5].

The cerebellum has been consistently related not only to motor control (coordination, static and dynamic balance, alternating rapid movements, and motor planning and timing) but also to the efficiency of memory, attention, visuospatial functions, language, and emotions, with a prominent role in functional integration with the cerebral cortex [6,7,8,9]. Moreover, the cerebellum could play a role in cognitive–motor interference (CMI), a concept that refers to the mutual influence that cognitive and motor functioning have on each other [10]. In MS patients, CMI impact appears increased [11].

Physiological and anatomical studies conducted on healthy adults and various clinical populations have revealed a topographic organization within the cerebellum, comprising distinct motor and cognitive regions [12]. Furthermore, recent research has demonstrated that these functionally specialized motor and cognitive areas of the cerebellum exhibit strong correlations with both motor and cognitive performance in pwMS, highlighting the cerebellum’s critical role in these processes [13]. Most recently, specific cerebellar lobules have been identified as being associated with the integration of motor and cognitive efficiency in MS patients [14]. Therefore, MS-related damage of the cerebellum and the cerebellar connections with other parts of the CNS (cortex, brainstem, and spinal cord) could significantly contribute to the development of disability and fatigue in this population [15].

Despite these findings, the role of the cerebellum in MS-related fatigue and disability is poorly understood. Therefore, in this study, we aimed to examine the correlation between fatigue, measures of clinical disability, and magnetic resonance imaging (MRI) of the cerebellum, hypothesizing that the different components of fatigue in MS patients may be related to altered volume during the traditional MRI and voxel-based morphometry (VBM) sequence, a whole-brain objective method that provides more sensitivity in comparing small-scale and regional differences in grey matter (GM) or white matter (WM) [16]. In particular, consistently with the functional topography (an anterior sensorimotor vs. posterior cognitive/emotional dichotomy) in the human cerebellum [17,18], we expected a correlation between physical fatigue and both the sensorimotor cerebellum (anterior lobe made up of lobules I–V; adjacent parts of lobule VI) and motor cerebellum (lobules VIIIA/B), between cognitive fatigue and the cognitive cerebellum (lobules VI and VII: VIIB, crus I, and crus II, lobule IX), and between psychosocial fatigue and the so-called “limbic cerebellum” (vermis, lobule VI, and left crus I, crus II) due to its connections with the cerebral limbic structures.

## 2. Materials and Methods

### 2.1. Study Design, Setting, and Ethical Approval

This cross-sectional study was conducted at the IRCCS “Santa Lucia” Foundation in Rome, Italy.

The protocol was conducted, recorded, and reported following the Good Clinical Practice guidelines and the Declaration of Helsinki [19] and approved by the Local Ethics Committee (CE/PROG.444-09, 28 April 2014). Before collecting data, all the participants signed an informed consent form.

### 2.2. Participants

To participate in the study, patients had to satisfy the following inclusion criteria: (1) have a diagnosis of MS according to revised McDonald’s criteria [20] with a relapsing-remitting (RR-MS) or secondary-progressive (SP-MS) disease course; (2) be 18–65 years old; (3) have an Expanded Disability Status Scale (EDSS) score ≤ 6.5 points [21]; and (4) be able to comprehend and carry out the planned assessments.

Exclusion criteria included primary-progressive (PP-MS) disease course, history of neurological diseases other than MS, and specific treatments for chronic fatigue and/or ataxia. Patients with cognitive impairment severity that interfered with the comprehension of tasks, severe systemic diseases, and a significant visual, auditory, or linguistic impairment or the presence of one of the strict contraindications to MRI execution (i.e., implanted electric and electronic devices such as heart pacemakers, especially older types, and insulin pumps, implanted hearing aids, neurostimulators, intracranial metal clips, and metallic bodies in the eye). Moreover, patients were excluded if they suffered from an MS relapse in the 3 months preceding their enrollment or if they received steroids over the previous month.

### 2.3. Clinical Assessment

To investigate the associations between CF and disability status and cerebellum, all participants underwent a clinical and radiological assessment. All participants were rated for the current level of disability with EDSS [21] by a certified neurologist or physiatrist with several years of experience. The EDSS is a scale that helps quantify MS disability and monitor changes in the level of disability over time.

Fatigue was assessed with the Modified Fatigue Impact Scale (MFIS) [22]. The MFIS is a 21-item-multidimensional patient-reported scale used to explore different aspects of fatigue on everyday functioning by analyzing its impact on physical, cognitive, and psychosocial domains. The global score of the MFIS (global-MFIS) is a combination of 10 items for cognitive status (cognitive MFIS, cMFIS), nine items for physical status (physical MFIS, pMFIS), and two items for psychosocial functional status (psychosocial MFIS, psMFIS). It is applied to classify the patients as fatigued (F: score of 38 or higher) or as non-fatigued (nF: score lower than 38) [23].

### 2.4. Radiological Assessment

A neuro-radiologist with several years of experience in the evaluation of pwMS performed MRI scans using a 3Tesla scanner equipped with a 32-channel magnetic imaging coil (Philips Achieva, Philips, Best, The Netherlands). The MRI images were acquired to measure the volume of each cerebellar region (total cerebellar grey matter (GM), vermis, and lobules). Additionally, a VBM sequence was applied to enable a voxel-by-voxel comparison of these regions.

**Figure 1:**
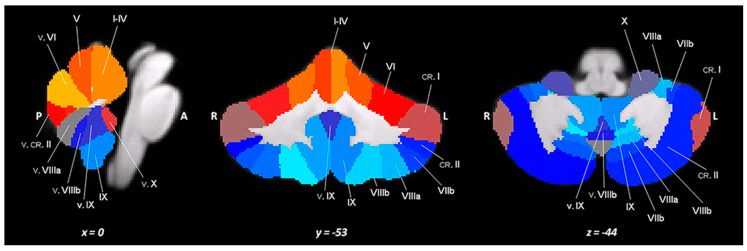
A spatially unbiased atlas template of the cerebellum and brainstem (SUIT).

A high-resolution T1-weighted MPRAGE sequence was acquired for each participant (repetition time (TR) = 11 ms; echo time (TE) = 5.3 ms; flip angle = 8°, matrix = 256 × 228 × 190; slab thickness = 0.9 mm; field of view (FOV) = 230 × 192 × 167 mm^3^). The analysis of the lobular volumes was carried out using the Spatially Unbiased Infra-tentorial Template (SUIT) probabilistic atlas (Figure 1), a GM template that excludes the white matter (WM) [24,25] and is implemented in Statistical Parametric Mapping (SPM8; www.fil.ion.ucl.ac.uk/spm, accessed on 20 December 2021). The procedure involved several phases: cutting out and isolating the cerebellum from the anatomical images in T1; normalizing each cut image in the SUIT space; superimposing the probabilistic cerebellar atlas on the individual space of the subject using the deformation parameters derived from normalization; and calculating the number of voxels in each lobule in each image. Therefore, this process resulted in 28 volumetric segments of GM, which reflected the bilateral and vermian lobules (Figure 1): lobules I–IV (right and left side) combined in a single measure; lobules V and VI (right and left side); lobule VII divided into VIIB (right and left side), crus I, and crus II (right and left side); and lobule VIII divided into VIIIA and VIIIB (both right and left side), lobule IX and X (both right and left side), and vermis (median parts of VI–X lobules). Finally, for each analyzed individual, the lobular volumes were normalized for the total cerebellar volume (individual lobular volumes/total cerebellar volume).

### 2.5. Statistical Analysis

All statistical analyses were run with IBM SPSS (Statistical Package for Social Sciences for Windows, Version 21.0; IBM Corp., Armonk, NY, USA) package software.

Descriptive statistics were computed to report the collected variables. Moreover, to study differences between F and n-F subgroups, an independent *t*-test was performed. Finally, correlations between conventional MRI and clinical variables were assessed using the Spearman rank correlation coefficient (ρ). Results were rated as follows: ρ > 0.70 = strong correlation, 0.50 < ρ < 0.70 = moderate correlation, and ρ < 0.50 = weak correlation [26].

A *p* < 0.05 was considered statistically significant.

The a priori hypotheses were stated as follows (a priori table is available as Supplementary Material—Table S1):

(1) The correlation between pMFIS and sensorimotor cerebellum volume was presumed to be negative and moderate (i.e., 0.50 < ρ < 0.70) because the sensorimotor cerebellum is involved in motor coordination, which can be impacted by physical fatigue. Similarly, the correlation between pMFIS and motor cerebellum volume was presumed to be moderate to strong and negative (i.e., 0.50 < ρ < 0.70 or ρ > 0.70), as motor cerebellum dysfunction is closely linked to physical fatigue;

(2) The correlation between cMFIS and cognitive cerebellum volume was presumed to be strong and negative (i.e., ρ > 0.70) because the cognitive cerebellum plays a critical role in higher-order cognitive functions that are significantly affected by cognitive fatigue;

(3) The correlation between psMFIS and limbic cerebellum volume was presumed to be moderate to strong and negative (i.e., 0.50 < ρ < 0.70 or ρ > 0.70) because the limbic cerebellum is involved in emotional and social processing, which are key components of psychosocial fatigue;

(4) The correlation between EDSS and cognitive/limbic cerebellum volume was presumed to be moderate and negative (i.e., 0.50 < ρ < 0.70) due to the cerebellum’s role in cognitive and emotional functions. Similarly, the correlation between disability and sensorimotor cerebellum volume was presumed to be moderate to strong and negative (i.e., 0.50 < ρ < 0.70 or ρ > 0.70), while the correlation with motor cerebellum volume was presumed to be strong and negative (i.e., ρ > 0.70), reflecting its role in motor control;

(5) For non-fatigued individuals, the correlation between physical fatigue (pMFIS) and sensorimotor/motor cerebellum volumes was presumed to be weak and negative (i.e., ρ < 0.50). Similarly, the correlation between cognitive fatigue (cMFIS) and cognitive/limbic cerebellum volumes was presumed to be weak and negative (i.e., ρ < 0.50);

(6) The correlation between psychosocial fatigue (psMFIS) and limbic cerebellum volume for non-fatigued individuals was presumed to be weak and negative (i.e., ρ < 0.50);

(7) Finally, for non-fatigued individuals, the correlation between disability (EDSS) and cognitive/limbic/sensorimotor cerebellum volumes was presumed to be weak and negative (i.e., ρ < 0.50), while the correlation with motor cerebellum volume was assumed to be moderate and negative (i.e., 0.50 < ρ < 0.70);

## 3. Results

### 3.1. Clinical and Structural MRI Measures

A total of 44 patients were included in this study. Table 1 summarizes the main demographic (sex, age, education), clinical (disease course, disease duration from the diagnosis, and EDSS and MFIS scores), and conventional MRI characteristics of the enrolled patients.

A total of 17 (39%) MS patients were classified as F-MS. Compared to non-fatigued MS (nF-MS) patients, F-MS patients were older (47.1 ± 10.2 vs. 41.7 ± 10.3 years), predominantly affected by secondary-progressive (SP-MS) disease course, and had similar years of education (14.3 ± 3.9 vs. years 14.5 ± 2.7 years), higher disease duration (13.8 ± 9.3 vs. 9.7 ± 9.8 years), and EDSS score (4.0 ± 2.0 vs. 3.0 ± 1.0 points). Moreover, F-MS patients presented lower volumes of sensorimotor cerebellum (3835.2 ± 570.2 vs. 4054.2 ± 654.1 mm^3^) in comparison with nF-MS patients.

### 3.2. Analysis of Correlations

The results of the correlation analysis between clinical and radiological parameters in the groups of MS patients are summarized in Table 2 (F-MS group) and Table 3 (nF-MS group).

In F-MS, as expected regarding the correlation between EDSS scores and MRI parameters, higher disability was correlated with cognitive and limbic cerebellum atrophy. Specifically, significant correlations were observed with atrophy in the VI lobule (left side: r = −0.580, *p* < 0.05; right side: r = −0.515, *p* < 0.05) and its normalized value by intracranial volume (LeftVI-ICV: r = −0.583, *p* < 0.05). Surprisingly, a direct correlation emerged with the vermis, part of the limbic cerebellum (VermisCrusI-ICV: r = 0.666; *p* < 0.01).

Moreover, as anticipated, pMFIS was inversely correlated with the volume of the sensorimotor lobules (pMFIS with right V lobule/total GM cerebellum ratio: r = −0.597, *p* < 0.05).

However, some peculiar correlations emerged. Although a negative correlation between psMFIS and the limbic cerebellum was initially hypothesized, a positive relationship was observed instead. Specifically, psMFIS scores were positively correlated with crus II (left side: r = 0.570, *p* < 0.05; right side: *p* = 0.511, *p* < 0.05) and with the normalized volume of the left crus Il adjusted for intracranial volume (LeftCruslI-ICV: r = 0.494, *p* < 0.05). Moreover, a positive association emerged between pMFIS and the limbic cerebellum (LeftCrusII-ICV: r = 0.514, *p* < 0.05). Furthermore, cMFIS was inversely related to the limbic cerebellum volume (cMFIS with the VermisVIIIb: r = −0.555, *p* < 0.05; with VermisVIIIb-ICV: r = −0.629 *p* < 0.01; with VermisVIIIb/total GM cerebellum ratio: r = −0.648, *p* < 0.01).

## 4. Discussion

This study aimed to investigate the potential correlation between fatigue and abnormalities in cerebellar volumes in pwMS. The findings indicate that fatigue in MS seems associated with specific patterns of cerebellar atrophy, with distinct relationships observed between the physical, cognitive, and psychosocial components of fatigue and different cerebellar subregions.

F-MS patients demonstrated reduced sensorimotor cerebellar volumes compared to nF-MS patients. Higher EDSS scores in F-MS patients were linked to atrophy in cognitive and limbic cerebellar regions. Physical fatigue was inversely correlated with sensorimotor cerebellum volume. Interestingly, a positive correlation was observed between EDSS scores and the vermis region of the limbic cerebellum. Notably, contrary to initial hypotheses, psychosocial fatigue was positively correlated with the volume of the limbic cerebellum, while cognitive fatigue showed a negative relationship with limbic cerebellum volume.

Functionally, multiple mechanisms contribute to the development of CF. These include hyperactivity resulting from maladaptive cortical reorganization and reduced functional connectivity caused by dysfunction and disruptions within motor and non-motor networks across various regions of the frontal, parietal, occipital, and temporal lobes as well as the cerebellum [27]. Additionally, abnormal adaptation and impaired timing in the recruitment of motor networks during prolonged effort further exacerbate the occurrence of CF [28]. Specifically, emerging evidence suggests the cerebellum’s critical role as a functional hub in CF. Studies have reported an association between cognitive fatigue (both state and trait forms) and hyperactivation in the caudate nucleus, prefrontal regions, and cerebellum as well. This heightened activation is thought to reflect the increased effort required by F-patients to perform even simple tasks [29].

Indeed, reduced movement-related brain activity has been observed in F-patients across various cortical and subcortical regions involved in motor planning and execution. These areas include the contralateral middle frontal gyrus, ipsilateral precuneus, and cerebellum as well as the contralateral thalamus [30]. A recent study by Hidalgo de la Cruz further corroborated these findings, demonstrating that F- patients exhibit thalamic resting-state functional connectivity (RS-FC) abnormalities with the middle frontal gyrus, sensorimotor network (SMN), precuneus, insula, and cerebellum. These connectivity disruptions were found to correlate with global MFIS scores [31]. Specifically, higher thalamic RS-FC with precuneus and lower RS-FC with posterior cerebellum as correlated with cognitive MFIS; higher thalamic RS-FC with sensorimotor network in frontal, motor, and temporal thalamic sub-regions was associated with physical and psychosocial MFIS, and reduced thalamic RS-FC with right insula in motor, postcentral, and occipital thalamic subregions was correlated with psychosocial fatigue.

Structurally, regional GM/WM atrophy, cortical thinning, decreased T1 relaxation times, and reduced fractional anisotropy were associated with central fatigue in MS. Total fatigue was related to alterations in the lower thalamic, striatum, pallidal, and superior cerebellar peduncle; instead, cognitive fatigue was related to extensive WM/GM atrophy of the occipital lobes, frontal lobe, parietal lobe, precuneus, primary sensorimotor area, supplementary motor area, bilateral precentral motor cortex, and brainstem [27].

Also, the study by Andreasen et al. on patients with progressive MS who underwent a cognitive and an MRI examination showed that the level of fatigue (assessed via the Fatigue Scale for Motor and Cognitive Function, FSMC) is related to the volume of specific cerebral areas. In greater detail, the authors found a correlation between the motor domain of fatigue and the regional cortical atrophy within the primary motor cortex and a trend toward a relationship between cognitive fatigue and the thickness of cortical areas involved in attentional processes [32].

Our findings align with previous studies examining the relationship between cerebral networks and fatigue scores assessed by scales such as the MFIS or FSMC. Specifically, our results suggest that both the physical and cognitive domains of CF are associated with atrophy in specific cerebellar subregions. Additionally, greater disability was found to correlate with atrophy in the cognitive and limbic regions of the cerebellum. As expected, the pMFIS was related to the sensorimotor cerebellum.

In contrast, cMFIS was associated with the limbic cerebellum, particularly vermis VIII and IX, rather than the cognitive cerebellum. Similar findings have been reported in studies on Parkinson’s disease (PD). In an observational study by Yin et al. investigating the role of the cerebellum in both motor and non-motor symptoms of PD patients with or without visuospatial disorders (VSD), vermis VIII, IX, and X demonstrated positive functional connectivity (FC) with the default mode network (DMN), executive control network, and sensorimotor network (SMN). Notably, PD patients with VSD exhibited significantly higher FC compared to controls between vermis VIII and whole-brain voxels as well as positive FC between vermis X and lobules VIII and IX of the left cerebellar hemisphere [33]. Considering that both MS and PD, due to their specific central nervous system (CNS) damage and associated neuronal loss leading to neurodegeneration, result in a reduced capacity for brain plasticity and adaptability when performing challenging single- or dual-task activities, parallelism could be drawn in explaining these findings [34]. Van Es et al. also reported strong functional connectivity between cerebellar vermis VIII and IX with the DMN, which may be involved in the integration of visual–spatial information and is related to visual motor coordination [35]. Thus, the cerebellar vermis VIII and IX may integrate visual–spatial information in PD patients. Therefore, this may represent compensatory activation of the cognitive network. As PD progresses, the function of the striatum–thalamus–cortex circuit worsens, and the cerebellum–thalamus–cortex circuit works harder to compensate for cognitive deficits.

This confirms the results of the study by Argento et al. on the role of the cerebellum in CMI in MS patients: in fact, in this study, the vermis VIIIa and IX also seemed to be involved in highly demanding cognitive tasks [14]. In MRI images, these specific regions of the cerebellum appear with higher volume than controls, probably due to a reorganization by dendritic arborization or augmented intraneural connections.

Therefore, the cerebellar vermis in MS patients also seems to modify the integrated networks for improved efficiency with fewer neural demands to achieve the same performance during dual tasking.

Lastly, understanding the relationship between psychosocial fatigue and the limbic cerebellum requires an examination of the multifactorial nature of fatigue in MS, the role of cerebellar atrophy, and the contribution of psychosocial factors to fatigue perception. Psychosocial fatigue in MS is often intertwined with cognitive and emotional impairments, leading to significant functional limitations in daily life [36]. The pathophysiology of fatigue in MS does not solely stem from physiological factors but also includes psychological and social dimensions that warrant consideration. Halicka et al. emphasized the complex interplay of biological, disease-related, behavioral, and psychosocial factors contributing to fatigue and depression among MS patients, thereby providing a comprehensive framework for understanding these experiences [37]. Moreover, selective cerebellar atrophy has been associated with fatigue and depression in MS patients [38]. Research indicates that structural changes in the cerebellum, including atrophy, are related to increasing fatigue levels [39]. Specifically, alterations in the cingulum bundle and its connection to cerebellar structures have been shown to mediate subjective feelings of fatigue [40]. The cerebellum’s involvement in both motor control and cognitive function suggests that dysfunction in this area can result in substantial psychological impacts, indirectly increasing the perception of fatigue in MS patients. Therefore, the positive association between MFIS-based psychosocial fatigue and limbic cerebellum volume can be attributed to both the direct impact of cerebellar dysfunction on cognitive and emotional health and the feedback loop between perceived fatigue and the psychosocial environment experienced by MS patients.

Our study opens a new perspective on the possible role of cerebellar rehabilitation on fatigue levels in MS patients. For instance, considering the relationship between physical fatigue (pMFIS) and sensorimotor cerebellum, a re-educational approach focused on physical and or/vestibular impairments could be beneficial. A study by Redlicka et al. supported the notion that moderate physical activity tailored to individual capabilities may enhance functional and emotional states while concurrently decreasing fatigue levels, suggesting that incorporating exercises that focus on balance and coordination into rehabilitation protocols could yield significant benefits [41]. On the same topic, García-Muñoz et al. demonstrated that vestibular rehabilitation combined with immersive virtual reality notably improved outcomes in fatigue-related assessments among MS patients, suggesting that better balance and coordination can mitigate fatigue impacts [42]. This aligns with findings from Abasi et al., who reported a direct correlation between vestibular rehabilitation and significant reductions in fatigue experiences within the MS population [43]. Furthermore, multimodal rehabilitation strategies have been highlighted for their potential to enhance overall well-being and specifically target fatigue. The systematic review conducted by Chasiotis et al. emphasized the role of cerebellar-focused therapy strategies, which can effectively engage multiple aspects of physical rehabilitation to manage symptoms such as fatigue. These strategies often involve integrating various therapeutic modalities, which have been proven beneficial for reducing fatigue levels [44]. Regarding psychosocial fatigue (psMFIS) and the cerebellum, Johansson et al. highlighted that lifestyle factors, including physical activity, could influence the impact of fatigue on psychosocial functioning. Their findings suggested that engaging in regular physical exercise ameliorates physical symptoms and plays a vital role in enhancing psychological well-being. This, in turn, may lead to a reduction in fatigue levels, emphasizing the importance of incorporating physical activity into care protocols for MS patients [45]. Notably, cognitive–behavioral therapy has been recognized as an effective intervention for managing fatigue-related symptoms. Gier et al. reported that CBT effectively reduces MS-related fatigue and improves overall psychological health. This supports the notion that a multifaceted rehabilitation approach encompassing cognitive and emotional support may yield better outcomes in managing fatigue in MS patients. Such interventions can complement cerebellar rehabilitation protocols by addressing the emotional fatigue that often accompanies neurological disorders [46].

### Limitations

Our study has some limitations. This is a cross-sectional study, so further studies should employ a longitudinal design to investigate the neural correlates of fatigue throughout the disease course. Another limitation of our study is the absence of objective motor and cognitive measures. However, this limitation is mitigated by the use of the MFIS, a validated self-reported scale, which still correlates with self-reported measures such as pMFIS and cMFIS. Moreover, we limited our analysis to a resting-state MRI. Last but not least, our sample size is low, which limits the generalizability of our results.

Future studies could overcome these limitations, including a higher number of clinical and radiological measures and expanding the sample size to achieve stronger results.

## 5. Conclusions

This study provides compelling evidence for the cerebellum’s involvement in fatigue and disability in pwMS. The findings highlight distinct correlations between cerebellar subregions and various fatigue components (i.e., physical, cognitive, and psychosocial) as well as clinical disability. These results underscore the cerebellum’s multifaceted role in integrating motor, cognitive, and emotional functions, which may be disrupted in MS-related fatigue. Moreover, our findings contribute to the growing body of literature emphasizing the cerebellum’s critical role as a functional hub in CF.

Future research should adopt longitudinal designs to track changes over time and incorporate objective motor and cognitive measures to complement self-reported scales. Additionally, advanced imaging techniques could further elucidate the functional connectivity and structural dynamics of cerebellar networks in pwMS. Such research could pave the way for targeted therapeutic strategies aimed at mitigating fatigue and improving QoL for pwMS.

## Figures and Tables

**Table 1:** Main demographic, clinical, and conventional MRI characteristics of the study groups.

	F-MS (n = 17)	nF-MS (n = 27)	*p*-Value
Sex			0.651 *
Men	7 (41.1%)	13 (48.1%)	
Women	10 (58.9%)	14 (51.9%)	
Age [years]	47.1 ± 10.2	41.7 ± 10.3	0.094 **
Education [years]	14.3 ± 3.9	14.5 ± 2.7	0.851 **
MS phenotype			0.001 *
Relapsing-remitting	5 (29.4%)	22 (81.5%)	
Secondary progressive	12 (70.6%)	5 (18.5%)	
Disease duration [years]	13.8 ± 9.3	9.7 ± 9.8	0.173 **
EDSS score	4.3 ± 1.7	2.7 ± 1.2	0.001 **
MFIS score	47.8 ± 9.7	20.4 ± 11.3	<0.001 **
MFIS physical score	24.1 ± 4.5	12.8 ± 6.1	<0.001 **
MFIS cognitive score	19.1 ± 6.8	5.4 ± 4.9	<0.001 **
MFIS psychosocial score	4.6 ± 1.7	2.2 ± 2.6	<0.001 **
T1 GMV TOTAL C [mm^3^]	116,182.8 ± 13,751.7	116,735.6 ± 11,545.8	0.886 **
T1 GMV SMC [mm^3^]	3835.2 ± 570.2	4054.2 ± 654.1	0.262 **
T1 GMV MC [mm^3^]	4392.1 ± 747.8	4207.3 ± 653.7	0.392 **
T1 GMV CC [mm^3^]	7625.0 ±3507.9	7667.4 ± 3611.0	0.969 **
T1 GMV LC [mm^3^]	4068.3 ± 4494.5	4112.7 ± 4543.7	0.974 **

Abbreviations: F-MS: fatigued multiple sclerosis patients; nF-MS: non-fatigued multiple sclerosis patients; MS, multiple sclerosis; EDSS: Expanded Disability Status Scale; MFIS: Modified Fatigue Impact Scale; GMV: gray matter volume; TOTAL C: total cerebellum; SMC: sensorimotor cerebellum (anterior lobe made up of lobules I–V; adjacent parts of lobule VI); MC: motor cerebellum (lobules VIIIA/B); CC: cognitive cerebellum (lobules VI and VII: VIIB, crus I and crus II, and lobule IX); LC: limbic cerebellum (vermis, lobule VI, and left crus I, crus II). **Notes:** Data are presented as frequency (percentage) or mean ± standard deviation. * Chi-square test; ** *t*-test for dependent samples.

**Table 2:** Correlation between MRI and clinical parameters in the F-MS group.

	EDSS	totMFIS	pMFIS	cMFIS	psMFIS
LeftVIGM	−0.580 *	−0.262	−0.176	−0.170	−0.110
RightVIGM	−0.515 *	−0.135	−0.084	−0.087	0.058
LeftCrusIIGM	−0.023	0.419	0.443	0.391	0.570 *
RightCrusIIGM	0.195	0.280	0.352	0.197	0.511 *
LeftVIIbGM	−0.028	0.264	0.276	0.264	0.509 *
VermisVIIIbGM	−0.050	−0.517 *	−0.124	−0.555 *	0.210
LeftV/totGMc	−0.211	−0.172	−0.417	0.010	−0.550 *
RightVGM/totGMc	−0.146	−0.248	−0.597 *	0.047	−0.442
LeftVIIb/totGMc	0.378	0.252	0.274	0.196	0.488 *
VermisVIIIb/totGMc	0.077	−0.549 *	−0.204	−0.648 **	−0.109
VermisIX/totGMc	−0.136	−0.391	0.039	−0.570 *	−0.144
LeftVI-ICV	−0.583 *	−0.214	−0.160	−0.167	−0.233
VermisCrusI-ICV	0.666 **	−0.088	0.010	0.000	0.366
LeftCrusII-ICV	0.303	0.386	0.514 *	0.204	0.494 *
VermisVIIIb-ICV	0.211	−0.460	0.014	−0.629 **	0.157

Abbreviations: F-MS: fatigued multiple sclerosis patients; EDSS: Expanded Disability Status Scale; totMFIS: total score of Modified Fatigue Impact Scale; pMFIS: physical Modified Fatigue Impact Scale; cMFIS: cognitive Modified Fatigue Impact Scale; psMFIS: psychosocial Modified Fatigue Impact Scale; GM: gray matter; totGMc: total GM cerebellum; ICV: normalization by use of intracranial volume. Notes: * *p* < 0.05; ** *p* < 0.01.

**Table 3:** Correlation between MRI and clinical parameters in the nF-MS group.

	EDSS	totMFIS	pMFIS	cMFIS	psMFIS
totMFIS	0.447 *	/	/	/	/
pMFIS	0.419 *	/	/	/	/
LeftCrusIGM	−0.416 *	−0.156	−0.145	0.002	−0.211
LeftVIIIaGM/totGMc	−0.014	−0.269	−0.173	−0.424 *	0.036
VermisXGM/totGMc	0.202	0.241	0.235	0.034	0.393 *
RightXGM/totGMc	0.408 *	−0.046	−0.151	0.033	0.204
LeftCrusIGM-ICV	−0.409 *	−0.171	−0.056	−0.188	−0.265
RightCrusIIGM-ICV	−0.091	−0.168	−0.021	−0.401 *	−0.074
LeftVIIbGM-ICV	−0.236	−0.245	−0.094	−0.458 *	−0.082
LeftVIIIaGM-ICV	−0.228	−0.298	−0.124	−0.504 **	−0.086
LeftVIIIbGM-ICV	−0.209	−0.277	−0.078	−0.469 *	−0.133
LeftIXGM-ICV	−0.218	−0.102	0.095	−0.390 *	−0.041
totGMc-ICV	−0.404 *	−0.222	−0.020	−0.417 *	−0.215

Abbreviations: nF-MS: non-fatigued multiple sclerosis patients; EDSS: Expanded Disability Status Scale; totMFIS: total score of Modified Fatigue Impact Scale; pMFIS: physical Modified Fatigue Impact Scale; cMFIS: cognitive Modified Fatigue Impact Scale; psMFIS: psychosocial Modified Fatigue Impact Scale; GM: gray matter; totGMc: total GM cerebellum; ICV: normalization by use of intracranial volume. Notes: * *p* < 0.05; ** *p* < 0.01.

## Data Availability

Data are available upon reasonable request to the corresponding author.

## Supplementary Materials

The following supporting information can be downloaded at: https://www.mdpi.com/article/10.3390/jcm14082840/s1, Table S1: Hypothesis testing between the fatigue component and the cerebellar substructure volume for fatigued and non-fatigued subgroups title.

## References

[1] Adams R.D., Victor M., Ropper A.H., Daroff R.B. (1997). Principles of Neurology. Neuropsychiatry Neuropsychol. Behav. Neurol..

[2] Chaudhuri A., Behan P.O. (2000). Fatigue and Basal Ganglia. J. Neurol. Sci..

[3] Khan F., Amatya B., Galea M. (2014). Management of Fatigue in Persons with Multiple Sclerosis. Front. Neurol..

[4] Cook K.F., Bamer A.M., Roddey T.S., Kraft G.H., Kim J., Amtmann D. (2013). Multiple Sclerosis and Fatigue: Understanding the Patient’s Needs. Phys. Med. Rehabil. Clin. N. Am..

[5] Bossa M., Manocchio N., Argento O. (2022). Non-Pharmacological Treatments of Cognitive Impairment in Multiple Sclerosis: A Review. NeuroSci.

[6] Schreck L., Ryan S., Monaghan P. (2018). Cerebellum and Cognition in Multiple Sclerosis. J. Neurophysiol..

[7] Schmahmann J. (1998). The Cerebellar Cognitive Affective Syndrome. Brain.

[8] Clausi S., Bozzali M., Leggio M.G., Di Paola M., Hagberg G.E., Caltagirone C., Molinari M. (2009). Quantification of Gray Matter Changes in the Cerebral Cortex after Isolated Cerebellar Damage: A Voxel-Based Morphometry Study. Neuroscience.

[9] Tedesco A.M., Chiricozzi F.R., Clausi S., Lupo M., Molinari M., Leggio M.G. (2011). The Cerebellar Cognitive Profile. Brain.

[10] Leone C., Feys P., Moumdjian L., D’Amico E., Zappia M., Patti F. (2017). Cognitive-Motor Dual-Task Interference: A Systematic Review of Neural Correlates. Neurosci. Biobehav. Rev..

[11] Tramontano M., Argento O., Manocchio N., Piacentini C., Orejel Bustos A.S., De Angelis S., Bossa M., Nocentini U. (2024). Dynamic Cognitive–Motor Training versus Cognitive Computer-Based Training in People with Multiple Sclerosis: A Preliminary Randomized Controlled Trial with 2-Month Follow-Up. JCM.

[12] Stoodley C.J., Schmahmann J.D. (2010). Evidence for Topographic Organization in the Cerebellum of Motor Control versus Cognitive and Affective Processing. Cortex.

[13] Fritz N.E., Edwards E.M., Ye C., Prince J., Yang Z., Gressett T., Keller J., Myers E., Calabresi P.A., Zackowski K.M. (2022). Cerebellar Contributions to Motor and Cognitive Control in Multiple Sclerosis. Arch. Phys. Med. Rehabil..

[14] Argento O., Spanò B., Pisani V., Incerti C.C., Bozzali M., Foti C., Caltagirone C., Nocentini U. (2021). Dual-Task Performance in Multiple Sclerosis’ Patients: Cerebellum Matters?. Arch. Clin. Neuropsychol..

[15] Weier K., Banwell B., Cerasa A., Collins D.L., Dogonowski A.-M., Lassmann H., Quattrone A., Sahraian M.A., Siebner H.R., Sprenger T. (2015). The Role of the Cerebellum in Multiple Sclerosis. Cerebellum.

[16] Mechelli A., Price C.J., Friston K.J., Ashburner J. (2005). Voxel-Based Morphometry of the Human Brain: Methods and Applications. Curr. Med. Imaging.

[17] Stoodley C., Schmahmann J. (2009). Functional Topography in the Human Cerebellum: A Meta-Analysis of Neuroimaging Studies. NeuroImage.

[18] Schmahmann J.D. (2019). The Cerebellum and Cognition. Neurosci. Lett..

[19] (2013). World Medical Association Declaration of Helsinki: Ethical Principles for Medical Research Involving Human Subjects. JAMA.

[20] Polman C.H., Reingold S.C., Banwell B., Clanet M., Cohen J.A., Filippi M., Fujihara K., Havrdova E., Hutchinson M., Kappos L. (2011). Diagnostic Criteria for Multiple Sclerosis: 2010 Revisions to the McDonald Criteria. Ann. Neurol..

[21] Kurtzke J.F. (1983). Rating Neurologic Impairment in Multiple Sclerosis: An Expanded Disability Status Scale (EDSS). Neurology.

[22] Fisk J.D., Ritvo P.G., Ross L., Haase D.A., Marrie T.J., Schlech W.F. (1994). Measuring the Functional Impact of Fatigue: Initial Validation of the Fatigue Impact Scale. Clin. Infect. Dis..

[23] Piscitelli D., Brichetto G., Geri T., Battista S., Testa M., Monti Bragadin M., Pellicciari L. (2024). Italian Adaptation and Psychometric Validation of the Fatigue Impact Scale (FIS) and Its Modified Versions in Adults with Multiple Sclerosis: A Rasch Analysis Study. Disabil. Rehabil..

[24] Diedrichsen J., Balsters J.H., Flavell J., Cussans E., Ramnani N. (2009). A Probabilistic MR Atlas of the Human Cerebellum. NeuroImage.

[25] Diedrichsen J. (2006). A Spatially Unbiased Atlas Template of the Human Cerebellum. NeuroImage.

[26] Munro B.H. (2005). Statistical Methods for Health Care Research.

[27] Arm J., Ribbons K., Lechner-Scott J., Ramadan S. (2019). Evaluation of MS Related Central Fatigue Using MR Neuroimaging Methods: Scoping Review. J. Neurol. Sci..

[28] Rocca M.A., Meani A., Riccitelli G.C., Colombo B., Rodegher M., Falini A., Comi G., Filippi M. (2016). Abnormal Adaptation over Time of Motor Network Recruitment in Multiple Sclerosis Patients with Fatigue. Mult. Scler..

[29] Genova H.M., Rajagopalan V., Deluca J., Das A., Binder A., Arjunan A., Chiaravalloti N., Wylie G. (2013). Examination of Cognitive Fatigue in Multiple Sclerosis Using Functional Magnetic Resonance Imaging and Diffusion Tensor Imaging. PLoS ONE.

[30] Filippi M., Rocca M.A., Colombo B., Falini A., Codella M., Scotti G., Comi G. (2002). Functional Magnetic Resonance Imaging Correlates of Fatigue in Multiple Sclerosis. Neuroimage.

[31] Hidalgo de la Cruz M., d’Ambrosio A., Valsasina P., Pagani E., Colombo B., Rodegher M., Falini A., Comi G., Filippi M., Rocca M.A. (2018). Abnormal Functional Connectivity of Thalamic Sub-Regions Contributes to Fatigue in Multiple Sclerosis. Mult. Scler..

[32] Andreasen A.K., Iversen P., Marstrand L., Siersma V., Siebner H.R., Sellebjerg F. (2019). Structural and Cognitive Correlates of Fatigue in Progressive Multiple Sclerosis. Neurol. Res..

[33] Yin K., Zhou C., Yin L., Zhu Y., Yin W., Lu Y., Liu B., Ren H., Xu Z., Yang X. (2021). Resting-State Functional Magnetic Resonance Imaging of the Cerebellar Vermis in Patients with Parkinson’s Disease and Visuospatial Disorder. Neurosci. Lett..

[34] McIsaac T.L., Fritz N.E., Quinn L., Muratori L.M. (2018). Cognitive-Motor Interference in Neurodegenerative Disease: A Narrative Review and Implications for Clinical Management. Front. Psychol..

[35] van Es D.M., van der Zwaag W., Knapen T. (2019). Topographic Maps of Visual Space in the Human Cerebellum. Curr. Biol..

[36] Marafioti G., Cardile D., Culicetto L., Quartarone A., Lo Buono V. (2024). The Impact of Social Cognition Deficits on Quality of Life in Multiple Sclerosis: A Scoping Review. Brain Sci..

[37] Halicka D., Tarasiuk J., Szczepański M., Krajewska A., Kułakowska A. (2017). Fatigue Syndrome, Depression and the Quality of Life in Patients with Multiple Sclerosis. Pielęgniarstwo Neurol. Neurochir..

[38] Lazzarotto A., Margoni M., Franciotta S., Zywicki S., Riccardi A., Poggiali D., Anglani M., Gallo P. (2020). Selective Cerebellar Atrophy Associates with Depression and Fatigue in the Early Phases of Relapse-Onset Multiple Sclerosis. Cerebellum.

[39] Powell D.J.H., Liossi C., Schlotz W., Moss-Morris R. (2017). Tracking Daily Fatigue Fluctuations in Multiple Sclerosis: Ecological Momentary Assessment Provides Unique Insights. J. Behav. Med..

[40] Pardini M., Bonzano L., Bergamino M., Bommarito G., Feraco P., Murugavel A., Bove M., Brichetto G., Uccelli A., Mancardi G. (2015). Cingulum Bundle Alterations Underlie Subjective Fatigue in Multiple Sclerosis. Mult. Scler..

[41] Redlicka J., Zielińska-Nowak E., Lipert A., Miller E. (2021). Impact of Moderate Individually Tailored Physical Activity in Multiple Sclerosis Patients with Fatigue on Functional, Cognitive, Emotional State, and Postural Stability. Brain Sci..

[42] García-Muñoz C., Cortés-Vega M.-D., Hernández-Rodríguez J.-C., Fernández-Seguín L.M., Escobio-Prieto I., Casuso-Holgado M.J. (2022). Immersive Virtual Reality and Vestibular Rehabilitation in Multiple Sclerosis: Case Report. JMIR Serious Games.

[43] Abasi A., Raji P., Friedman J.H., Hadian M.-R., Hoseinabadi R., Abbasi S., Baghestani A. (2020). Effects of Vestibular Rehabilitation on Fatigue and Activities of Daily Living in People with Parkinson’s Disease: A Pilot Randomized Controlled Trial Study. Park. Dis..

[44] Chasiotis A.K., Kitsos D.K., Stavrogianni K., Giannopapas V., Papadopoulou M., Zompola C., Paraskevas G.P., Bakalidou D., Giannopoulos S. (2023). Rehabilitation on Cerebellar Ataxic Patients with Multiple Sclerosis: A Systematic Review. J. Neurosci. Res..

[45] Johansson S., Skjerbæk A.G., Nørgaard M., Boesen F., Hvid L.G., Dalgas U. (2021). Associations between Fatigue Impact and Lifestyle Factors in People with Multiple Sclerosis—The Danish MS Hospitals Rehabilitation Study. Mult. Scler. Relat. Disord..

[46] Houniet-de Gier M., Beckerman H., Van Vliet K., Knoop H., De Groot V. (2020). Testing Non-Inferiority of Blended versus Face-to-Face Cognitive Behavioural Therapy for Severe Fatigue in Patients with Multiple Sclerosis and the Effectiveness of Blended Booster Sessions Aimed at Improving Long-Term Outcome Following Both Therapies: Study Protocol for Two Observer-Blinded Randomized Clinical Trials. Trials.

