# Supplementary material for: The Role of the Cerebellum in Multiple Sclerosis-Related Fatigue and Disability
Source: J Clin Med. 2025 Apr 20;14(8):2840. doi: 10.3390/jcm14082840 (PMC12027523; doi:10.3390/jcm14082840)
Supplement: Supplementary file 1 [file jcm-14-02840-s001.zip › jcm-3582515-supplementary.pdf]

Table S1. Hypothesis testing between the fatigue component and the cerebellar substructure volume for fatigued and non-fatigued subgroups.

SUBGROUP	FATIGUE COMPONENT/DISABILITY	CEREBELLAR SUBSTRUCTURE VOLUME	EXPECTED HYPOTHESIS (DIRECTION AND STRENGTH OF CORRELATION)
Fatigued (F)	Physical Fatigue (pMFIS)	Sensorimotor cerebellum	Negative and moderate ($0.50<\varrho<0.70$)
		Motor cerebellum	Negative and moderate to strong ($0.50<\varrho<0.70$ or $\varrho>0.70$)
	Cognitive Fatigue (cMFIS)	Cognitive cerebellum	Negative and strong ($\varrho>0.70$)
	Psychosocial Fatigue (psMFIS)	Limbic cerebellum	Negative and moderate to strong ($0.50<\varrho<0.70$ or $\varrho>0.70$)
	Disability (EDSS)	Cognitive cerebellum	Negative and moderate ($0.50<\varrho<0.70$)
		Sensorimotor cerebellum	Negative and moderate to strong ($0.50<\varrho<0.70$ or $\varrho>0.70$)
		Motor cerebellum	Negative and strong ($\varrho>0.70$)
Non-Fatigued (nF)	Physical Fatigue (pMFIS)	Sensorimotor cerebellum	Negative and weak ($\varrho<0.50$)
		Motor cerebellum	Negative and weak ($\varrho<0.50$)
	Cognitive Fatigue (cMFIS)	Cognitive cerebellum	Negative and weak ($\varrho<0.50$)
	Psychosocial Fatigue (psMFIS)	Limbic cerebellum	Negative and weak ($\varrho<0.50$)
	Disability (EDSS)	Cognitive/limbic cerebellum	Negative and weak ($\varrho<0.50$)
		Sensorimotor cerebellum	Negative and weak ($\varrho<0.50$)
		Motor cerebellum	Negative and moderate ($0.50<\varrho<0.70$)

Abbreviations: MFIS: Modified Fatigue Impact Scale; pMFIS: physical MFIS; cMFIS: cognitive MFIS; psMFIS: psychosocial MFIS; EDSS: Expanded Disability Status Scale; Y: yes; N: no.
